# Thermochemical oxidation of methane induced by high-valence metal oxides in a sedimentary basin

**DOI:** 10.1038/s41467-018-07267-x

**Published:** 2018-12-03

**Authors:** Wen-Xuan Hu, Xun Kang, Jian Cao, Xiao-Lin Wang, Bin Fu, Hai-Guang Wu

**Affiliations:** 1 State Key Laboratory for Mineral Deposits Research, School of Earth Sciences and Engineering, Nanjing University, Nanjing, 210023 China; 2 Research School of Earth Science, The Australian National University, Canberra, ACT 0200 Australia

## Abstract

Thermochemical oxidation of methane (TOM) by high-valence metal oxides in geological systems and its potential role as a methane sink remain poorly understood. Here we present evidence of TOM induced by high-valence metal oxides in the Junggar Basin, located in northwestern China. During diagenesis, methane from deeper source strata is abiotically oxidized by high-valence Mn(Fe) oxides at 90 to 135 °C, releasing ^13^C-depleted CO_2_, soluble Mn^2+^ and Fe^2+^. Mn generally plays the dominant role compared to Fe, due to its lower Gibbs free energy increment during oxidation. Both CO_2_ and metal ions are then incorporated into authigenic calcites, which are characterized by extremely negative δ^13^C values (−70 to −22.5‰) and high Mn content (average MnO = 5 wt.%). We estimate that as much as 1224 Tg of methane could be oxidized in the study area. TOM is unfavorable for gas accumulation but may act as a major methane sink in the deep crustal carbon cycle.

## Abstract (web summary)

The role of thermochemical oxidation of methane (TOM) by high-valence metal oxides as a potential methane sink remains poorly understood. Here, the authors present evidence of TOM induced by Mn(Fe) oxides in a sedimentary basin, which yields calcite with extremely negative δ^13^C values (−70 to −22.5‰, VPDB).

## Introduction

Methane (CH_4_) is an economically important fossil fuel, and by 2014 about 4.3 × 10^14^ m^3^ or 4.7 × 10^5^ Tg of proven natural gas reserves, including shale gas, had been identified in sedimentary basins worldwide^1,2^. However, methane is also a significant greenhouse gas, second only to CO_2_ in the contribution to the current global greenhouse effect^3,4^; during certain intervals of the geologic past, such as the Archean, it was perhaps the most significant greenhouse gas in the atmosphere^5,6^. Since the late Holocene pre-industrial era, the concentration of atmospheric methane had increased sharply from an average of 680 p.p.b.v. (parts per billion volume) during AD 800–1600 to 1799 p.p.b.v. by the year 2010^4,7^. Furthermore, the current emission flux of methane from sedimentary basins to the atmosphere is estimated to be 10 to 25 Tg yr^−1^, which is a key to understanding the global carbon cycle and climate change^8,9^.

Oxidation reactions are the key mechanism by which methane is consumed before emission into the atmosphere. Methane can theoretically be oxidized via two distinct geological processes. The first is anaerobic oxidation of methane (AOM), where methane oxidation is mediated by sulfate- or metal-reducing bacteria in an anoxic environment, typically resulting in the precipitation of authigenic carbonate cements^10–12^. The second is thermochemical oxidation of methane (TOM) at high temperatures by sulfate or high-valence metal oxides. Like the bacterially-mediated AOM reaction, this process typically results in the formation of authigenic carbonate^13–16^.

Natural gas reservoirs in sedimentary basins are typically buried to depths of several kilometers, and experience temperatures from 60 to 150 °C^17^, while the metabolism of the archaeal groups responsible for AOM are mainly active at temperatures below 80 °C^18,19^, limiting the depth at which archaea dominate the conversion of CH_4_ to CO_2._ Therefore, TOM is potentially the predominant methane oxidation process in the strata with high formation temperature (especially > 80 °C) ^13–15,20^.

Thermochemical sulfate reduction (TSR) consuming methane has been observed to occur in geological systems^13–15,20^. Its reaction conditions, as well as geological and geochemical responses, have been deeply explored^13–15,20,21^. In contrast, almost no geological evidence has been reported for the oxidation of methane with high-valence metal oxides, except for the oxidation of methane by Fe^3+^ in detrital biotite at temperatures above 270 °C during metamorphism^16,22,23^. To date, the process has only been simulated in the laboratory setting at high temperatures (350 to 650 °C) ^24–27^. Thus, both the extent of TOM by high-valence metal oxides in geological systems and its role in the methane sink remain poorly understood.

**Fig. 1:**
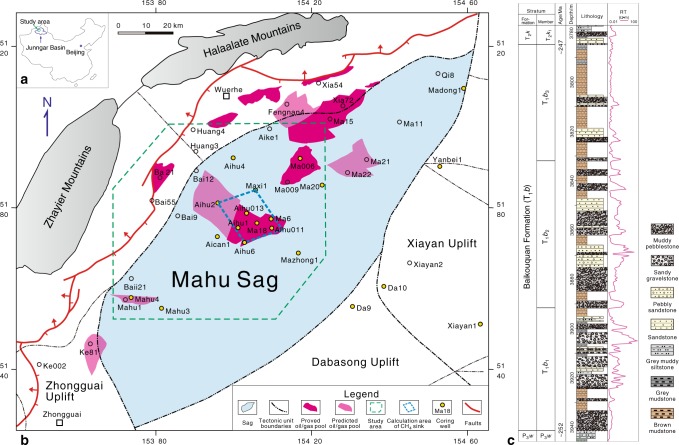
Schematic geologic map of the Mahu Sag, and generalized stratigraphy of the Lower Triassic Baikouquan Formation. **a** The location of the Junggar Basin in China. **b** The study area is located in the west slope of the Mahu Sag adjacent to the northwestern marginal thrust faults of the Junggar Basin. **c** The sun-shape symbols within the Resistivity logging column indicate reservoir beds rich oil and gas

The Junggar Basin is a major petroliferous basin in northwestern China, with an area of approximately 1.3 × 10^5 ^km^2^ ^28^. The Lower Triassic Baikouquan Formation (T_1_*b*) is located on the western slope of the Mahu Sag, a structure forming part of the northwestern margin of the Junggar Basin (Fig. 1a). The Mahu Sag is the richest hydrocarbon reservoir in the Junggar Basin, with approximately two billion tons of proven crude oil reserves^29^. Continuous oil and gas reservoirs were recently discovered in the Baikouquan Formation (Fig. 1a), with the hydrocarbons believed to be derived largely from the dark, organic-rich mudstones of the underlying Lower Permian Fengcheng Formation (see Supplementary Note 1 and Supplementary Fig. 1 for detailed descriptions)^28,30^.

The Baikouquan Formation can be divided into three members, referred to as T_1_*b*_1_, T_1_*b*_2_, and T_1_*b*_3_ in ascending stratigraphic order. The maximum burial depth of the unit from west to east across the Mahu Sag increases from 2850 m to 4300 m. The formation consists of interlayered mudstone and sandy conglomerate, deposited in a succession of alluvial fan-deltaic systems with abundant gravity flow deposits^31,32^. The brown or greyish-green conglomeratic layers contain pebble conglomerates, sandy gravel conglomerates, and sandstones, whereas the mudstone layers are dominated by massive brown silty mudstones with minor gray laminations (Fig. 1b). The abundance of brown mudstone increases up-section in three members, which is interpreted to reflect an arid-semiarid climate regime and oxic lacustrine environment during deposition^31,33^.

The T_1_*b* sediments are derived from two provenances, i.e., the basement, comprising granite and mafic-ultramafic igneous rocks, and the sedimentary strata from the Carboniferous to the Permian^28,31^. The weathering of mafic-ultramafic rocks and tuffaceous components in sedimentary rocks forms abundant high-valence Fe-Mn oxides during T_1_*b* deposition interval (see Supplementary Note 2 for provenance analysis)^34^.

In this study, we report the first geological evidence for extensive TOM induced by high-valence metal oxides in the Lower Triassic reservoir strata of the Junggar Basin. We find that methane was abiotically oxidized by high-valence Mn(Fe) oxides at high temperatures (90–135 °C) in the deep burial strata, ultimately yielding extremely ^13^C-depleted authigenic calcites with high manganese contents. In the process thousands of Tg of CH_4_ were consumed, implying TOM may act as a major CH_4_ sink in the deep carbon cycles.

## Results

### Distribution and geochemistry of calcite cements

**Fig. 2:**
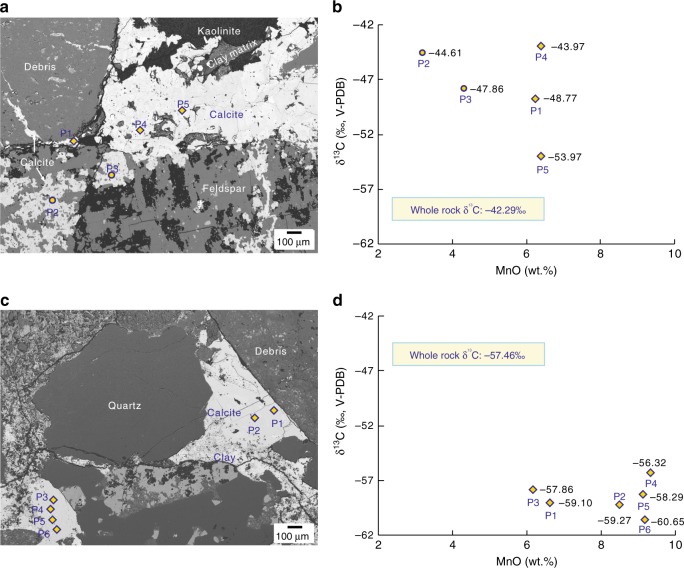
Back scattered electron microscopic images of sandy conglomerates from the Baikouquan Formation, with crossplots of in situ δ^13^C and Mn contents of authigenic calcite. **a**, **c** Sandy conglomerate from the T_1_*b*_3_ member (3824.2 m in Well M18), with two generations of calcite cement. The early-stage cements have consistently lower MnO content than the late-stage cements. **b**, **d** Sandy conglomerate from the T_1_*b*_2_ member (3866.9 m in Well M18), cemented by calcite with higher MnO content. Circles indicate measurements of early-stage calcite, diamonds represent measurements of late-stage cements. The overall values of late-stage calcite δ^13^C are lower than those of the early-stage calcite. The error bars, referring to the standard error of the mean (SEM) in this paper, is smaller than the symbol size in the cross-plots

Authigenic calcite in the sandy conglomerates of the Baikouquan Formation occurs mainly as coarsely crystalline cement (Fig. 2a, b), sometimes within feldspar along cleavage (Fig. 2a). Based on microscopic observations, the total content of calcite cement in the sandy conglomerates ranges from 0 to 6% by area, with an average of ~2.5%. There are two stages of precipitation: the early-stage calcite occurs as xenomorphic crystals filling dissolved pores or poorly-connected interparticle pores (Fig. 2a), whereas the late-stage cements appear as coarse crystals, with well-developed crystal planes in the central portions of large interparticle pores (Fig. 2a, b).

**Fig. 3:**
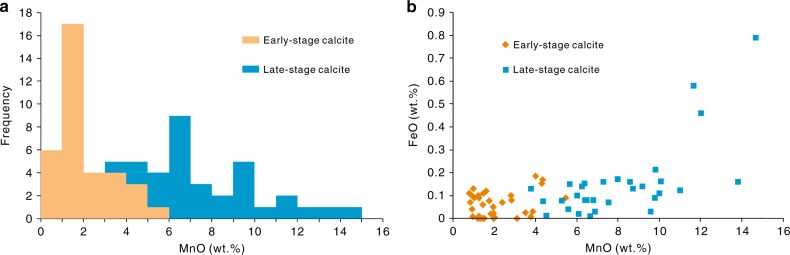
Manganese and iron contents in authigenic calcites. **a** Histogram of MnO content in early-stage and late-stage calcite cements from T_1_*b* sandy conglomerates. **b** Crossplot of MnO and FeO content in early-stage and late-stage calcite cements from T_1_*b* sandy conglomerates. The error bar (±SEM) is smaller than the symbol size. Source data are provided as Supplementary Data 1

Electron probe micro-analysis (EPMA) reveals that these authigenic calcites are enriched in manganese, with MnO content ranging from 0.79 to 14.67 wt.% (average = 5.05 wt.%; Fig. 3a). In detail, the MnO content of early-stage calcite cements is generally lower than 4.00 wt.%, while late-stage cements typically have MnO content greater than 5.00 wt.%, some up to 11.00–15.00 wt.% (Fig. 3a). In contrast, the FeO content of the calcite cements is very low, ranging from<0.01 to 0.79 wt.%, with an average of 0.12 wt.% (Fig. 3b).

**Fig. 4:**
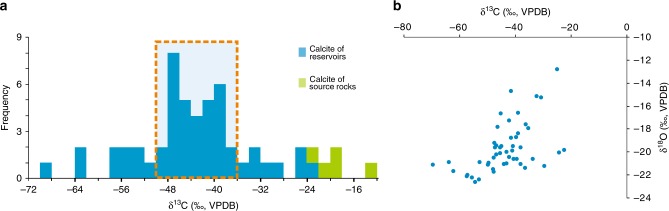
Carbon and oxygen isotopic compositions in authigenic calcites. **a** Histogram of δ^13^C values, of calcite cements in T_1_*b* reservoirs, and calcite veins in P_2_*w* source rocks. **b** Crossplot of bulk rock δ^13^C and δ^18^O values in T_1_*b* calcite cements. The error bar (±SEM) is smaller than the symbol size. Source data can be seen in Supplementary Data 2

Authigenic calcite cements in the T_1_*b* reservoir rocks are very negative in δ^13^C. Whole-rock carbonate δ^13^C values range from −69.8 to −22.5‰, though most samples fall within the −50.0 to −36.0‰ range (Fig. 4a). The calcite cements have δ^18^O values ranging from −22.6 to −12.8‰ (Fig. 4b).

In situ carbon isotope analyses reveal that the two generations of calcite cements have markedly different δ^13^C values, sometimes by 10‰ (Fig. 2c, d). In contrast, the difference in δ^18^O between the two generations of cement is relatively small, with a maximum offset of 1.9‰ (Supplementary Table 1). To better constrain the sources of the carbon and oxygen incorporated into calcite cements in the T_1_*b* reservoir, we also measured the isotopic composition of calcite in the underlying source rocks: −22.4 to −13.5‰ for δ^13^C; and −18.5 to −12.8‰ for δ^18^O.

### Geochemistry of hydrocarbons

In most natural gas samples more than 95 mol. % hydrocarbons are light components, i.e., C_1_–C_7_. CH_4_ is the most abundant hydrocarbon gas, with an aridity coefficient of 0.77–0.95. The non-hydrocarbon component consists mainly of N_2_, with minor CO_2_ and no detectable H_2_S. The ranges of δ^13^C values in methane, ethane, propane, and butane are −39.3 to −46.8‰, −27.5 to −31.9‰, −27.2 to −30.2‰, and −26.0 to −28.8‰, respectively (Supplementary Table 2).

**Fig. 5:**
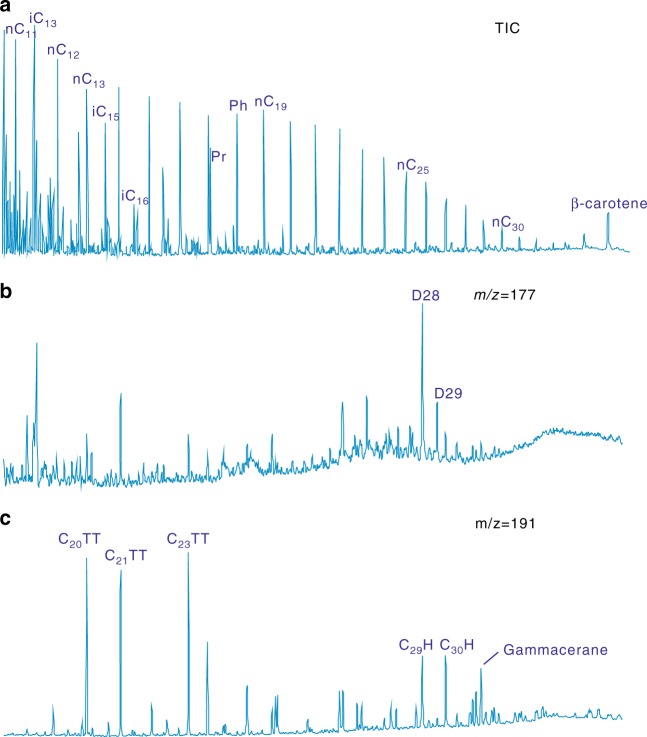
Chromatogram and mass spectrogram of a representative T_1_*b* crude oil sample (No. M18-O-1). **a** In the total ion chromatogram (TIC) Pr and Ph indicate pristane and phytane, respectively. **b** D represents demethylated hopanes in the *m*/*z* 177 mass spectrogram. **c** TT is tricyclic terpane and H is hopane in the *m*/*z* 191 mass spectrogram

Gas chromatography of crude oil samples from T_1_*b* reveals a complete sequence of n-alkanes (Fig. 5), especially ~C_8_–C_12_ which are preferentially removed in the earliest stages of biodegradation^19^. This indicates that the hydrocarbons have not experienced microbial degradation. The absence of 25-norhopanes and other representative values of key parameters also show no evidence for biodegradation (Supplementary Table 3). Biomarker analyses are described in details in the Methods section.

## Discussion

The extremely negative δ^13^C values of authigenic calcite can be interpreted to be derived from organic matter^35,36^. In organic matter-enriched geological systems, both oxidation of organic matter and thermal decarboxylation of organic acids can generate significantly ^13^C-depleted CO_2_, which may then be incorporated into authigenic calcite^11–13,27,35^. The δ^13^C value of authigenic calcite formed via the oxidation of hydrocarbons can be as low as −125‰^11–13,36^, whereas calcite precipitated from the decarboxylation of organic acids concentrates from −3 to −25‰, rarely yielding values below −25‰^35,37^. Authigenic calcite in the study area shows δ^13^C values falling mainly into the range of −50.0 to −36.0‰, with a minimum value of −69.8‰, consistent with the oxidation model for gaseous hydrocarbons^11,12,36^.

**Fig. 6:**
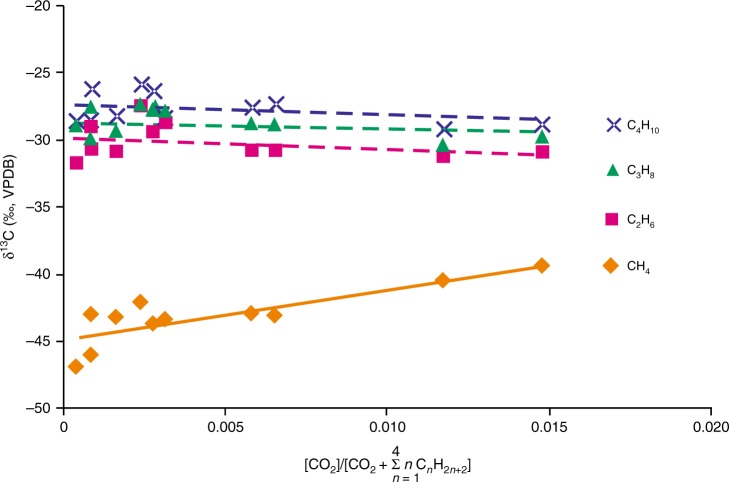
Carbon isotopes of natural gases from T_1_*b* sandy conglomerate reservoirs plotted against [CO_2_]/[CO_2_ + ∑_*n*=1_*n*C_*n*_H_2*n*+2_]. The latter is a parameter estimating the extent of thermochemical sulfate reduction^39^. The error bar (±SEM) is smaller than the symbol size

**Fig. 7:**
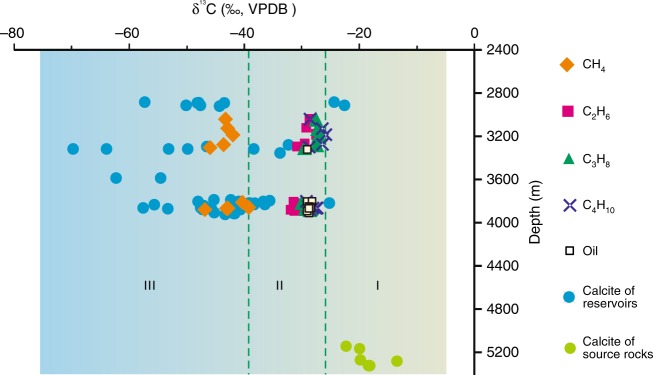
Carbon isotopes of natural gas, calcites from reservoir strata, and calcite veins from source rocks. Region I represents carbonates associated with decarboxylation of organic acids, while Region III denotes carbonates derived from the oxidation of methane. Region II indicates carbonates associated with a combination of the oxidation of methane and small quantities of C_2+_, as well as decarboxylation. The error bar (±SEM) is smaller than the symbol size

During the oxidation of gaseous hydrocarbons, ^12^C is preferentially oxidized to CO_2_, leaving the remaining hydrocarbon pool to become increasingly enriched in ^13^C as the reaction proceeds^24,38,39^. Figure 6 shows that the δ^13^C value of methane in T_1_*b* natural gas samples increases from around −45‰ to values above −40‰ as the CO_2_ contents of the gas increases; however, the isotopic composition of heavier hydrocarbon gases (C_2_–C_4_) remains comparatively stable. This result demonstrates that, within the T_1_*b* natural gas pool, abundant CH_4_ was oxidized during diagenesis. The δ^13^C values of the authigenic calcite in T_1_*b* are generally lower than those of the associated methane (−46.8 to 39.3‰; Fig. 7), further supporting our interpretation that the calcite is mainly derived from CO_2_ generated by methane oxidation^11,12,36^.

There are two mechanisms for oxidization of methane in sedimentary systems, i.e., AOM and TOM^10,11,13–15^. AOM has been observed mainly in modern marine sediments, especially near cold seeps on the seafloor, where the δ^13^C values of the associated authigenic carbonate can be as low as −125‰^10,11,36^. TOM can occur with or without TSR reactions. Methane is usually not the most active reductant in systems where TSR occurs compared with C_2+_ gaseous alkanes, so the minimum δ^13^C value of the associated calcite is not usually lower than −31‰^13,15,39,40^. TOM reactions induced by high-valence metal oxides instead of sulfate have never been reported in natural systems, though they have been simulated in the laboratory^24–27^.

All the three processes described above may have contributed to the precipitation of calcites in T_1_*b*. The range of measured δ^13^C values (−69.8 to −22.5‰) is compatible with calcite formed via AOM, but is also lower than the typical value of calcite formed via methane-dominated TSR (δ^13^C = −31‰)^13^. While there are no carbon isotope data reported for the carbonate associated with TOM induced by high-valence metal oxides in geological systems, the fractionation factors may be assumed to be similar to those in laboratory-simulated experiments. Thus, we cannot rule out the TOM process based on our isotopic measurements.

Anaerobic oxidation of methane by bacteria or archaea can be ruled out. Firstly, although archaeal groups such as ANME-1 and ANME-2 can anaerobically metabolize methane in the presence of oxidants such as MnO_2_, SO_4_^2−^, and Fe^3+^ at temperatures < 80 °C^10,18,19,41^, the high formation temperature (> 80 °C) does not favor the metabolism of these archaeal groups^19^. The T_1_*b* reservoir strata were buried at depths of 2250–3200 m when they were firstly charged with methane in the Early Jurassic^32^. Taking the geothermal gradient as 28–32 °C km^−1^ and the surface temperature as 20 °C during the Early Jurassic^42^, this corresponds to a formation temperature of 83–122 °C. The δ^18^O values of authigenic calcites also indicate precipitation of the cements at relatively high temperatures. A similar diagenetic condition was reported for the Brent Group of the North Sea with a burial depth of 2500–3500 m^43,44^. The δ^18^O value of T_1_*b* pore water is likely to have increased by the same value (about 4‰) as the Brent Group from that of the initial meteoric water (−10 to −8‰) to −6 to −4‰^44–46^. Using this δ^18^O value, we calculated that the temperature was greater than 90 °C during formation of more than 85% of the total calcite, with a background temperature range of 90–135 °C and an average of 109.6 °C (see Supplementary Data 2), consistent with the temperatures estimated from the geothermal gradient.

Secondly, T_1_*b* hydrocarbons show no evidence of biological degradation, indicating that archaea had negligible influence on the composition of oil and gas. The first manifestation of hydrocarbon biodegradation is typically the selective removal of C_8_–C_12_ normal alkanes^19^. Light C_2_–C_6_ hydrocarbons exhibit a consistent degradation sequence: propane is the first compound to be altered, followed by butane, pentane, and C_6+_ hydrocarbons^47^. The net effect of biodegradation is an overall decrease in wet gas components (C_2+_) and enrichment in methane^19^. The crude oil hosted in the Baikouquan Formation shows a complete alkane series, with no obvious loss of C_8_–C_12_ alkanes, and no hump of unresolved complex mixtures (Fig. 5). Natural gas samples contain 5.14–22.68% wet gas (C_2+_) and 0.79–5.23% propane. Because AOM depends on the presence of biological mediators, and there is no evidence for biological modification, we can rule out this process of methane oxidation^19,47^.

Thermochemical sulfate reduction can also be ruled out. There is very little SO_4_^2−^ in T_1_*b* formation waters^48^, and no H_2_S has been detected in T_1_*b* natural gas samples (Supplementary Table 2) or the associated crude oil in adjacent formations^28,29^. Pyrite is also absent in T_1_*b* reservoir strata. The lack of both key reactants (i.e., sulfate) and characteristic products (sulfide in gaseous or mineral forms) strongly suggests that the TSR reaction did not occur in our study area. Thus, the highly negative δ^13^C values seen in the authigenic calcites cannot be attributed to methane oxidation by sulfate.

Therefore, we infer that the extremely negative δ^13^C observed in the study area results from methane oxidation by non-sulfate oxidants at high temperatures. It is shown that methane can serve as an electron acceptor for high-valence Mn and Fe oxides, leading to the formation of CO_2_ and the precipitation of calcite^10^. This process can be expressed with the following chemical formulas:

1 $${\mathrm{CH}}_{\mathrm{4}}{\mathrm{ + 4Mn}}_{\mathrm{2}}{\mathrm{O}}_{\mathrm{3}}{\mathrm{ + 15H}}^{\mathrm{ + }} \to {\mathrm{HCO}}_{\mathrm{3}}^{\mathrm{ - }}{\mathrm{ + 8Mn}}^{{\mathrm{2 + }}}{\mathrm{ + 9H}}_{\mathrm{2}}{\mathrm{O}}\quad\Delta {{G = -442}}\,{\mathrm{kJ/mol}}$$

2 $${\mathrm{CH}}_{\mathrm{4}}{\mathrm{ + 4MnO}}_{\mathrm{2}}{\mathrm{ + 7H}}^{\mathrm{ + }} \to {\mathrm{HCO}}_{\mathrm{3}}^{\mathrm{ - }}{\mathrm{ + 4Mn}}^{{\mathrm{2 + }}}{\mathrm{ + 5H}}_{\mathrm{2}}{\mathrm{O}}\quad\Delta {{G = -556}}\,{\mathrm{kJ/mol}}$$

3 $${\mathrm{CH}}_{\mathrm{4}}{\mathrm{ + 8Fe}}\left( {{\mathrm{OH}}} \right)_{\mathrm{3}}{\mathrm{ + 15H}}^{\mathrm{ + }} \to {\mathrm{HCO}}_{\mathrm{3}}^{\mathrm{ - }}{\mathrm{ + 8Fe}}^{{\mathrm{2 + }}}{\mathrm{ + 21H}}_{\mathrm{2}}{\mathrm{O}}\quad\Delta {{G = -270}}{\mathrm{.3}}\,{\mathrm{kJ/mol}}$$

4 $${\mathrm{Ca}}^{{\mathrm{2 + }}}{\mathrm{ + HCO}}_{\mathrm{3}}^{\mathrm{ - }}{\mathrm{ + OH}}^{\mathrm{ - }} \to {\mathrm{CaCO}}_{\mathrm{3}} \downarrow {\mathrm{ + H}}_{\mathrm{2}}{\mathrm{O}}$$

The Gibbs free energy increment (Δ*G*) differs substantially between the first two reactions and the third one under the same conditions, with oxidation by Mn_2_O_3_ or MnO_2_ being approximately twice as energetically favorable as oxidation by Fe(OH)_3_^10^. In geological systems where high-valence Mn and Fe coexist, CH_4_ can be preferentially oxidized by Mn rather than Fe. During the interval of T_1_*b* deposition, the northwestern margin of the Junggar Basin experienced an arid or semiarid climate^31,33^. Mafic-ultramafic rocks were weathered extensively from the provenance during the period of deposition and were ultimately deposited in the organic matter-depleted red layers of T_1_*b* (Supplementary Fig. 2). These red beds provided the material basis for subsequent methane oxidation by the high-valence Mn and Fe oxides^49^. XRF analysis shows that the Mn content of sandy conglomerates in T_1_*b* ranges from 0.07 to 0.48 wt.% (as Mn_2_O_3_) in bulk rock, with an average of 0.17 wt.%; the Fe content ranges from 2.88 to 12.71 wt.% (as Fe_2_O_3_), with an average of 6.25 wt.% (Supplementary Table 4). Further XRD, FE-SEM, and EPMA analyses indicate that high-valence Mn(Fe) exists as hematite in the clastic rocks in T_1_*b*. Most of the hematite is disseminated in the clay-rich matrix, while minor occurrences as isolated amorphous aggregates are observed. The manganese generally occurs as the isomorphous Mn^3+/4+^ substitution in the hematite lattice structure^50,51^ at levels of 0.95–1.47 wt.% in the isolated hematite and 0.61–0.74 wt.% in the disseminated hematite in the form of Mn_2_O_3_ (see Supplementary Note 3 and Supplementary Fig. 3 for detailed descriptions).

The authigenic calcites in T_1_*b* are generally enriched in manganese relative to whole rock samples, with MnO content ranging from 0.79 to 14.67 wt.% (average = 5.05 wt.%). However, the FeO content of the calcite cements is uniformly less than 1 wt.%, averaging at 0.12 wt.%. This reflects the energetic favorability of Mn as an oxidant relative to Fe. Thus, the precipitation of calcite was closely associated with methane oxidation by high-valence manganese.

It should be noted that C_2+_ gaseous alkanes (especially ethane and propane) are usually preferentially oxidized during TSR reactions, whereas methane is the last to react^14,15,21,40^. The oxidation of abundant methane in T_1_*b* rocks has been influenced by several factors. One is the sufficient supply of Mn^3+/4+^, which is present as hematite within brown clastic rocks and guarantees that almost all of the gaseous alkanes, including methane, will be consumed in the bleaching pathways^49^. The other is that since methane is the main component of the T_1_*b* natural gas, ultimately it will have dominated the thermochemical oxidation process. Moreover, the T_1_*b* clastic strata are low-permeability due to their high clay content and complex grain composition^32^. Consequently, gaseous alkanes mainly migrated by diffusion. Due to its higher initial concentration and higher diffusion coefficient, the quantity of methane reaching the matrix of reservoir rocks or mudstones at a distance from the oil-gas interface is much higher than that of C_2+_ gaseous alkanes^52^. Ultimately, as shown in Fig. 6, abundant methane was oxidized in the T_1_*b* clastic rocks.

Microbial metabolism and high temperature can both serve to facilitate reactions (1) and (2). The former may reduce the energy threshold, while the latter can provide the activation energy necessary for the reaction to occur^10,24^. Since microbial activity in the T_1_*b* reservoir strata appears to have been minimal, heating would have been the only way to promote oxidation reactions. Considering the formation temperature (90–122 °C) during infiltration of natural gas and the main precipitation temperature (90–135 °C) of authigenic calcites, we can infer that methane oxidation in the study area mainly took place at 90–135 °C. Laboratory experiments have shown that reactions (1) and (2) do not occur at temperatures below 300 °C and 250 °C, respectively^24,27^. However, during extended geological processes, long intervals of time can compensate for the slow rate of reactions at low temperatures. In the case of thermochemical sulfate reduction, experiments show that the reaction occurs at temperatures greater than 250 °C^53^, though evidence for TSR has been observed in sedimentary basins at temperatures of ~100 °C^40^. As another example, while the standard Rock Eval pyrolysis procedure shows the peak of hydrocarbon generation at 420–480 °C, in natural geological systems the peak of hydrocarbon generation typically occurs at 120–150 °C^54^. This also appears to be the case for TOM. The long duration of geological time can compensate for a low reaction rate at relatively low temperatures. With the increasing burial depth of T_1_*b*, the formation temperature generally increased and reached 90 °C in the Middle Jurassic (~170 Ma, see Supplementary Note 4 and Supplementary Fig. 4 for thermal evolution). Since then, methane has been oxidized more efficiently.

**Fig. 8:**
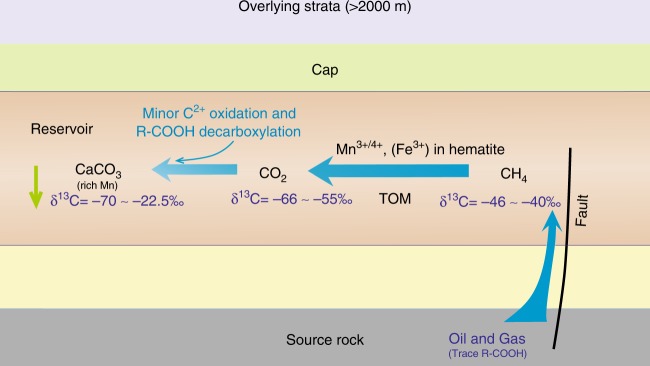
Schematic model of thermochemical methane oxidation in the clastic strata of petroliferous basins. R-COOH represents carboxylic acids. High-valence Mn (Fe) exists in hematite, which is generally found in the matrix of sandstone and conglomerate. Due to the high diffusivity of methane, minor high-valence Mn (Fe) in the brown-red mudstone adjacent to the reservoir rocks may also be reduced

Carbon isotope fractionation is expected to occur during thermal oxidation of methane, with the fractionation factor controlled by a number of factors, including the type of oxidant and the temperature^24,27,53^. In general, the residual methane is enriched in ^13^C as the reaction proceeds, while the generated CO_2_ is depleted, resulting in CO_2_ with a lower δ^13^C value than the initial methane pool (δ^13^C_i-CH4_). Taking the carbon isotope kinetic fractionation factor (*α*) to be 10^3^/(10^3^−∆) in reactions (1) and (2), where ∆ = δ^13^C_i-CH4_−δ^13^C_CO2_, we calculate an *α* value of 1.0175–1.0193 for Reaction (2) at 90–135 °C^24^. This value is close to that reported for the methane-dominated TSR in the East Sichuan Basin of China (1.0166) and the ethane-dominated TSR in the Jurassic Norphlet Formation of Mobile Bay (1.109)^55^. Then, a ∆ value of 17–19‰ for a closed system at 90–135 °C can be obtained. Carbon isotope fractionation also occurs during the calcite precipitation. At 90 to 135 °C, the δ^13^C of precipitated CaCO_3_ is expected to be 2 to 5‰ lower than that of the original gaseous CO_2_^56^. Integrating the methane oxidation and calcite precipitation processes, the δ^13^C of authigenic calcite is expected to be 19 to 24‰ lower than that of the residual methane. Thus, the expected δ^13^C of authigenic calcite is −70 to −59‰. The extremely ^13^C-depleted calcites may also precipitate during the oxidation of primary microbial or kerogen-cracking-derived low δ^13^C methane, although their relative quantities involved in the reaction remain ambiguous. The former is formed via bacterial methanogenesis at temperatures generally below 50–60 °C in the P_1_*f* source rock^57^, whereas the latter is generated as a concomitant of liquid oil expelled from low-maturity source rock^19^. In addition, it should be noted that the contributions of oxidation of minor C_2+_ and decarboxylation of organic acids from the source rock cannot be ruled out (Fig. 8). As shown in Fig. 7, the decarboxylation reaction can produce CO_2_ with a δ^13^C value of around −25‰. The addition of small amounts of CO_2_ from C_2+_ oxidation and decarboxylation yields a range of potential δ^13^C values in authigenic calcite from −70 to −22.5‰, consistent with our observations (Fig. 8).

Methane is generally believed to be stable in the crust, especially in sedimentary basins; oxidation is thought to occur mainly after the methane has reached the surface and been exposed to atmospheric oxygen^8,9^. The potentially important role of TOM as a methane sink in the deep strata of sedimentary basins has largely been neglected so far; methane oxidization in the deep crust has not been documented, and only a few relevant simulation experiments have been conducted to date^24,27,53^. In this study, we provide the first evidence for the occurrence of TOM in a clastic reservoir, and propose that TOM is an important CH_4_ sink in the deep crustal carbon cycle.

In order to evaluate the extent to which TOM serves as a CH_4_ sink, we made a series of semi-quantitative estimates based on the M18-Aihu2 oil producing area (highlighted in yellow in Fig. 1a), which contains a relatively dense concentration of exploration wells. Details of the calculations are described in the Methods section. Assuming an average authigenic calcite content of 2.5% in the relevant reservoir beds, we calculated a total authigenic calcite mass of 420 Tg. The calcite would require 57 Tg of CH_4_ to have been oxidized, assuming only 85% of the carbonate carbon is derived from methane oxidation. If only taking account of the total oil-charging area in the sag, a fairly conservative CH_4_ consumption is 155 Tg. However, authigenic calcites are not restricted in the conglomerates and sandstones charged by liquid oil, they also occur in the wells far away from the oil-charging area. Extending these calculations to encompass the entire Mahu Sag (~5000 km^2^), we estimate that as much as 1224 Tg of CH_4_ might be oxidized in the reservoir strata. The global submarine CH_4_ flux to the atmosphere is only 8 to 65 Tg per year^58^, suggesting that TOM in sedimentary basins may play a geologically significant role in reducing CH_4_ seepage to the surface. In other words, extensive TOM may be an important methane sink in sedimentary basins, while it is unfavorable for the accumulation of recoverable natural gas reserves.

This study reports geological evidence for widespread thermochemical oxidation of methane (TOM) induced by high-valence metal oxides in a natural environment, from the Junggar Basin in northwestern China. Methane was oxidized at high temperatures (90–135 °C) in the T_1_*b* stratum, with high-valence metal oxides acting as the electron acceptor. This reaction resulted in the precipitation of authigenic calcite cements, characterized by extremely negative δ^13^C values (−70 to −22.5‰) and high manganese contents (average MnO = 5 wt.%). While both Mn and Fe oxides served as methane oxidants, less Fe oxide was consumed due to the relatively higher Gibbs free energy increment of the reaction. Based on the abundance and distribution of authigenic calcite, we estimate that thousands of Tg of CH_4_ were consumed by TOM in the Lower Triassic reservoir strata of the Mahu Sag. We further propose that the geologic importance of TOM has been underestimated to date, and that this critical methane sink should be a focus of future studies of the deep carbon cycle.

## Methods

### Sampling

A total of 103 rock samples were collected from T_1_*b* reservoir beds in 11 wells from the western slope area of the Mahu Sag (Supplementary Table 5). In addition to rock samples, 9 crude oil and 12 natural gas samples were collected from the wells (Supplementary Table 6).

### Elemental analyses

Backscattering electron (BSE) microprobe analyses were conducted on 38 sandy conglomerate samples, using a Japan Electron Optics Laboratory (JEOL) x-ray analyzer (JXA)-8800 electron microprobe operated at 15 kV, with a 10 nA beam current. The calcite cements in 14 sandy conglomerate samples were analyzed for their major element composition, using a 2 μm beam diameter and 10 s counting time. The major element compositions of whole-rock samples were measured on a Thermo Scientific ARL 9900 × -ray fluorescence (XRF) spectrometer. The relative standard deviation of measured major element concentrations is around ± 1% for elements with concentrations > 1.0 wt.%, and about ± 10% for the elements with concentrations < 1.0 wt.%, based on repeated measurements of Chinese national standards GBW-07103 (granite) and GBW-07105 (basalt). Both EPMA and XRF analyses were carried out at the State Key Laboratory for Mineral Deposits Research, Nanjing University.

### Carbonate isotope analyses of bulk rock samples

The calcite cements in 50 T_1_*b* sandy conglomerate (i.e., reservoir rock) samples and 5 P_2_*w* mudstone (i.e., source rock) samples were analyzed for stable carbon and oxygen isotopes. Prior to analysis, each sample was broken into small pieces, and ~ 1 g of calcite cement was manually selected under a binocular microscope. All samples were then crushed to a fine powder. Twenty milligrams of each powdered sample was reacted with 100% H_3_PO_4_ at 25 °C for more than 12 h, in a Kiel IV autosampler (ThermoFinnigan). The produced CO_2_ was then introduced into a Finnegan MAT 253 mass spectrometer for isotopic analysis. Carbon and oxygen isotope measurements were corrected using Chinese national standard GBW 04405 (δ^13^C = 0.57 ± 0.03‰ VPDB; δ^18^O = −8.49 ± 0.13‰ VPDB), an Ordovician carbonate from a site near Beijing. All analyses were performed at the Nanjing Institute of Geology and Paleontology, Chinese Academy of Sciences. The external precisions (1σ) of oxygen and carbon isotope analyses were ± 0.08 and ± 0.03‰, respectively.

### SIMS δ^13^C_calcite_ analyses

Small pieces of polished thin sections of carbonate samples were mounted in Buehler EpoFix epoxy resin, together with carbonate reference materials: NBS-19 calcite, UWC-3 calcite (δ^13^C_VPDB_ = –0.91 ± 0.08‰, 2σ, *n* = 9; *cf*. ref. ^59^.), UW6220 dolomite (δ^13^C_VPDB_ = 0.84 ± 0.02‰, 2σ, *n* = 5; *cf*. ref. ^60^.) and Hammerfall dolomite (δ^13^C_VPDB_ = –0.28 ± 0.07‰, 2σ, *n* = 3; Ian S. Williams, personal communication). Backscattered electron images were taken using a JEOL JSM-6610A Analytical Scanning Electron Microscope in Research School of Earth Science (RSES), Australian National University (ANU), Canberra.

Secondary ion mass spectroscopy (SIMS) analyses of carbon isotopes were performed in situ using the Sensitive High Resolution Ion Microprobe for Stable Isotopes (SHRIMP SI) located in RSES, ANU. The analytical procedure was similar to ref. ^61^ for oxygen isotopes, though without electron-induced secondary ionization emission (EISIE) correction at the presence of the electron gun. Carbon isotopes (^12^C and ^13^C) were measured simultaneously by two Faraday cups, using an ~15 kV Cs + primary ion beam focused over an area of ~30 μm in diameter and source slit of 60 µm. ^12^CH was completely resolved with collector slits of 200 µm for ^13^C and 300 µm for ^12^C.

Duplicate δ^13^C analyses of the calcite standard UWC-3 yield an external (spot to spot) precision (1σ) of 0.40–0.50‰. Mn content (shown as Mn/(Ca + Mg + Fe + Mn); Supplementary Table 1) in the calcite samples is as high as 4.6–12.7 mol. %. Such small amount of Mn has less measurable matrix effects on SIMS δ^13^C bias than Fe^2+^ ^60^. Contamination of measurements by organic carbon was avoided by carefully choosing spots free of micro fractures or inclusions. Thus the potential error associated with matrix effect and organic inclusions is considered to be insignificant relative to isotopic variation.

### SIMS δ^18^O_calcite_ analyses

The oxygen isotope composition of calcite was measured using a SHRIMP II ion microprobe at the RSES facility of ANU. Analytical methods were similar to those described by ref. ^61^. Sequences of sample analyses (typically three measurements) were bracketed by 1–2 analyses of standards. Corrected ^18^O/^16^O ratios are reported in standard δ^18^O notation, relative to Vienna Pee Dee Belemnite (VPDB). All δ^18^O values were calibrated against the UWC-3 calcite standard (δ^18^O = −17.87‰, 1σ = 0.03‰; *cf*. ref. ^62^). The spot-to-spot reproducibility (i.e., external precision) was typically better than ± 0.30‰ (1σ).

### Composition and δ^13^C of natural gas

The composition of natural gas was determined using a Hewlett Packard 6890 II gas chromatograph (GC). Stable carbon isotope values were measured on an Optima isotope ratio mass spectrometer (IR-MS) coupled to a Hewlett Packard 6890 II GC. Measurements followed the procedure described by ref. ^29^. An internal CO_2_ reference gas was introduced into the spectrometer prior to and after the peaks of interest. Measured δ^13^C values were calibrated using the national ‘charcoal black’ standard, with a value of −22.43‰ relative to VPDB. All gas samples were analyzed in triplicate.

### Biomarkers and δ^13^C in oil samples

The biomarker compositions of oil samples were analyzed on a Hewlett Packard 6890 II GC, using a Hewlett Packard PONA capillary column with dimensions of 50 m × 0.25 mm × 25 μm. Stable carbon isotope analyses of alkanes and arenes were performed on the same instrument, following the procedure described by ref. ^63^.

### Estimating the methane sink capacity of the Mahu Sag

We first take the Mahu18-Aihu2 district, highlighted in blue dashed line in Fig. 1a, to be representative of the broader Mahu Sag. At least eight cores have been drilled in the 234 km^2^ area, providing a good overview of the T_1_*b* reservoir strata. The combined thickness of the horizons with secondary calcite cements ranges from 19.6 m to 48.3 m, with an average value of 28.7 m. Thus, the volume of rock containing secondary calcite can be estimated as: 234 × 10^6 ^m^2^ (area of the region of interest) × 28.7 m (average thickness of relevant horizons) = 6.7 × 10^9 ^m^3^ of calcite cement-bearing strata. Multiplying by a typical sedimentary rock density of 2.5 × 10^3^ kg m^-3^ converts this volume to a total mass of 16.8 × 10^12 ^kg.

Based on the microscopic observations, the content of calcite cement in the relevant strata ranges from 1% to 5%, with the average value of 2.5%. Thus, we can calculate the total mass of authigenic calcite to be: 16.8 × 10^12^ kg × 2.5% = 420.0 × 10^9^ kg of calcite cement.

With the above estimate of the total mass of calcite cement in the Mahu18-Aihu2 district, we can calculate the mass of methane that would have been necessary to generate this cement. Due to its diffusion superiority of volatile natural gas relative to liquid oil, it can be approximately assumed that all carbon in the cement is derived from natural gas. As the average relative content of CH_4_ is 85% in the T_1_*b* natural gas (Supplementary Table 2), the methane consumption can be estimated as: 420.0 × 10^9^ kg of CaCO_3_ ÷ 100.09 g mol^−1^ CaCO_3_ × 16.06 g mol^−1^ × 0.85 CH_4_ = 57.3 × 10^9^ kg (57.3 Tg) of CH_4_. Converting this mass to volume yields: 57.3 × 10^9^ kg CH_4_ ÷ 0.72 kg m^−3 ^≈ 79.6 × 10^10^ m^3^ of CH_4_.

If only taking account of the oil-charging area, a fairly conservative methane consumption is 155 Tg, as the total discovered oil-charging area has reached 633 km^2^ to the end of 2017 in the Mahu Sag (Fig. 1a). However, authigenic calcites are not restricted in the conglomerates and sandstones charged by oil, they also occur in the wells far away from the oil-charging area. The entire sag has an area of approximately 5000 km^2^, which is roughly 20 times greater than the area of the Mahu18-Aihu2 district. Thus, we can estimate that the total mass of CH_4_ abiotically oxidized by Fe-Mn oxides in the sag reaches 1,224 Tg.

The parameters used in the mass balance calculations are as follows: Mass_CH4_ = 16.05 g mol^−1^; Mass_CaCO3_ = 100.09 g mol^−1^; Density of CH_4_ = 0.72 kg m^−3^ (defined at 273.15 K and 101.325 kPa); Density of rock = 2.50 × 10^3^ kg m^−3^.

## Electronic supplementary material

Supplementary Information

Peer Review File

Description of Additional Supplementary Files

Supplementary Data 1

Supplementary Data 2

## Data Availability

All necessary data generated or analyzed during this study are included in this published article, and other auxiliary data are available from the corresponding authors on reasonable request.
