# Supplementary material for: Thermochemical oxidation of methane induced by high-valence metal oxides in a sedimentary basin
Source: Nat Commun. 2018 Dec 3;9:5131. doi: 10.1038/s41467-018-07267-x (PMC6277441; doi:10.1038/s41467-018-07267-x)
Supplement: Supplementary file 1 — Supplementary Information [file 41467_2018_7267_MOESM1_ESM.pdf]

Thermochemical oxidation of methane induced by high-valence metal oxides in a sedimentary basin

Hu et al.

Supplementary Notes

Supplementary Note 1: Geological outline of T_1b petroleum reservoirs

The basement of the Junggar Basin comprises granite and mafic–ultramafic volcanic rocks that formed at *ca* 800 Ma in the Precambrian[1]. Sedimentation started from the late Carboniferous, mainly as volcanic and clastic sequences, and gradually developed into a foreland basin. During the early–middle Permian, dark mudstones and clastic rocks interbedded with volcaniclastic layers were deposited, including the source-rock sequences of the study area; i.e., the Jiamuhe Formation (P_1j), the Fengcheng Formation (P_1f), and the Lower Wuerhe Formation (P_2w) [2] (Supplementary Fig. 1). In the late Permian, the Hercynian orogeny triggered the uplift of the northwestern part of the basin, producing a series of high-angle thrust faults. The basin then developed into an intracontinental lacustrine basin by the Early Triassic, filled with various clastic rocks including conglomerates, sandstones, and mudstones[3]. During the late Triassic, a set of mudstone sequences with sandstone interlayers, the Baijiantan Formation (T_3b), was deposited extensively as regional cap rocks. Sedimentation gradually ceased while the margins of the basin were still compressed, and the Upper Triassic deposits were overlain unconformably by Jurassic sequences. During the Late Jurassic to Cretaceous, the basin began to contract into a rejuvenated foreland basin as a result of the Yanshannian orogeny[4]. After the Cretaceous, sedimentation in the study area ceased except for some small-scale fluvial fans adjacent to the piedmonts of the Zhayier and Hala'alate mountains.

The Mahu Sag lies adjacent to the northwestern margin of the basin, with a length of ~120 km from north to south, and a width of ~40 km. Within the sag, Carboniferous to Permian strata are distributed across the entire study area, with Triassic to Cretaceous strata thinning gradually from the depocenter toward the marginal fault belts. The depth of the Triassic T_1b Formation gradually

increases from west to east, from 2800 m to 4400 m, while its thickness increases from 70 m to 180 m. The T_1b Formation unconformably overlies the Upper Wuerhe Formation (P_3w) of the late Permian, and is conformably overlain by the Karamay Formation (T_2k; Supplementary Fig. 1).

Since 2010, five large reservoirs of light oil (>100 Mt) have been discovered in sandy conglomerates of the T_1b Formation, with maximum oil and gas production rates of 48.6 t d^{-1} and 6900 m^3 d^{-1}, respectively[5-7]. Oil and gas were generated mainly in the Fengcheng Formation (P_1f) source rocks[2-5], and charging the T_1b reservoir rocks during the Early Jurassic and Early Cretaceous[8]. The oil and gas migrated directly from the P_1f Formation into the T_1b Formation sandy conglomerates along steeply dipping faults, and then continued to migrate through high-permeability sandy conglomerate units of the T_1b Formation[5, 7, 9] (Supplementary Fig. 1).

Supplementary Note 2: Sedimentary Environment and Provenance

The study area is adjacent to the Zhayier and Hala'alate mountains, which were uplifted on the northwest margin of the Junggar Basin. The Baikouquan Formation comprises mainly coarse clastic sedimentary rocks (mainly brown pebbly conglomerates) that were deposited in alluvial fans close to the western piedmont area. The sediments gradually become finer-grained over time; e.g., gravelly conglomerates, sandstones, and mudstones were deposited in retreating shallow lacustrine fan deltas in the sag areas[10, 11]. Detailed core logging indicates that this formation was a fan delta complex comprising multiple rock types and characterized by abundant gravity-flow deposits. This formation comprises five rock types, namely muddy pebbly conglomerates (debris-flow deposits), sandy gravelly conglomerates (subaqueous-channel and gravel-flow deposits), sandstone and pebbly sandstone (subaqueous-channel deposits), grey muddy siltstone (turbidite deposits), and brown silty

mudstone (interchannel flood deposits)[6]. Calcareous mudstones and sandstones, and playa sediments such as dolomite and gypsum do not occur in T_1b and adjacent formations. These features distinguish the T_1b Formation from the non-marine Triassic redbeds of NW Europe and the Triassic–Jurassic redbed sequences of North America, which comprise calcretes and playa lake sediments[12-15].

Successive global atmospheric-CO_2 greenhouse episodes with abnormally high temperatures occurred during the Early Triassic[16, 17], and the T_1b Formation contains sedimentary assemblages that are typically produced by debris flows, sheet floods, and high-energy stream flows, indicating that it was deposited in an arid–semiarid paleoclimate[10]. The unusually warm paleoclimate might have resulted in the depletion of organic matter during deposition, as recorded by abundant subsurface brown mudstones and conglomerates of the formation (Supplementary Fig. 2).

Shan et al.[18] investigated paleo-current directions based on analyses of dip-meter logging data, indicating that the provenance of the T_1b Formation includes paleo-uplifts along the northwestern margin of the basin. Further petrological analysis indicates that conglomerates in the study area are composed mainly of clasts of tuff, sedimentary rock, and granite, while coarse-grained sandy components are dominated by quartz, feldspar, and granite rock fragments[10, 18]. There was no carbonate supply to the sediments of the T_1b Formation. These observations indicate that sediments of the T_1b Formation were derived from two provenance areas with different rock types, namely the basin basement comprising granite and mafic–ultramafic igneous rocks, and underlying Carboniferous to Permian sedimentary sequences[2].

The weathering of mafic–ultramafic igneous rocks in the basin basement, and tuffaceous components in the Carboniferous and Permian sedimentary rocks, formed abundant high-valence Fe–Mn oxides before deposition of the T_1b Formation.

Supplementary Note 3: Occurrence of high-valence Mn(Fe) oxides

As mentioned Supplementary Note 2, the T_1b Formation comprises conglomerate, sandstone, and mudstone. The gravel-sized clasts of the conglomerate are mainly mafic–ultramafic tuff, sedimentary rock, and granite, while the sand-sized grains are dominated by quartz, feldspar, and granite rock fragments[6, 10, 18]. The matrix among the conglomeratic and sandy grains is mainly smectite and mixed-layer illite/smectite (I/S), with minor kaolinite and hematite (Supplementary Fig. 3a). In the mudstones, silty quartz, tuff, and granite rock fragments occur with various clay minerals such as smectite and I/S. Minor hematite is disseminated throughout the clay. Common heavy minerals (e.g., ilmenite, leucosphenite, rutile, and epidote) also occur in various rocks in the T_1b Formation[18].

XRD, FE-SEM, SEM-EDS, and EPMA analyses were performed on the brown mudstones and rown pebbly conglomerate from the T_1b reservoir sequences. Contrary to earlier assumptions, Mn does not occur as MnO_2 (e.g., pyrolusite, ramsdellite) but exists rather in hematite and Fe-rich chlorite, and in Mn-rich calcite. Hematite and iron-rich chlorite occur in the mudstones or in the matrix among grains of sandstones and conglomerate clasts. Most of the hematite is disseminated in the clay-rich matrix, with some occurring as isolated amorphous aggregates in the matrix (Supplementary Figs. 3a, b). The Mn_2O_3 content of the isolated hematite is 0.95–1.47 wt.% (average = 1.14 wt.%), and 0.61–0.74 wt.% (average = 0.68 wt. %) in the hematite–smectite mixture (Supplementary Fig. 3c), with the former being higher overall. The MnO content of the Fe-rich chlorite is 0.22–1.31 wt.% (average = 0.86 wt.%) [6].

Manganese valence was investigated by *in situ* X-ray photoelectron spectroscopy (XPS). Useful Mn 2p and 3s signals were not obtained due to the low Mn content[19, 20], but the reliable Fe 2p spectra

indicate that Fe is trivalent in hematite (Supplementary Fig. 3d). The reduction potentials of Mn^{3+} and Mn^{4+} are higher than that of Fe^{3+} [21, 22], so both Mn^{3+} and Mn^{4+} can oxidize Fe^{2+} to Fe^{3+}. Therefore, in the presence of Fe^{3+}, Mn generally occurs as an isomorphous $Mn^{3+/4+}$ substitution in hematite[23-26]. Where Fe^{2+} is present in the chlorite, Mn is expected to be bivalent as Fe^{2+} substitution[27].

In weakly acidic fluid, $Mn^{3+/4+}$ is released into formation water through the dissolution of hematite[28, 29]. The reduction of $Mn^{3+/4+}$ also promotes its dissolution and release. However, aqueous Mn^{3+} is unstable and tends to undergo disproportionation into Mn^{2+} and MnO_2 [29, 30]. In contrast to insoluble MnO_2 (oxidation potential, Eh = 1.224 V), aqueous Mn^{3+} (Eh = 1.542V) is characterized by a stronger oxidization capacity[21]. This may be why methane can be oxidized at such low temperatures.

Supplementary Note 4: Burial history and Diagenesis

Tectonic activity has not resulted in significant uplift of the Mahu Sag since the Early Triassic[8]. The depth of the Baikouquan Formation generally increased after deposition (Supplementary Fig. 4). However, the geothermal gradient decreased gradually during geological history, from 36.3 ℃ km^{-1} in the late Permian to 33.8 ℃ km^{-1} in the Late Triassic, 28.4 ℃ km^{-1} in the Late Jurassic, 24.8 ℃ km^{-1} in the Late Cretaceous, and eventually dropping to 22.8 ℃ km^{-1} in the Neogene, which is similar to the present geothermal gradient[22]. The thermal evolution of the T_1b Formation is indicated by its geothermal gradient and burial history. During the first period of methane charging (J_1), the formation temperature had reached above 83 ℃, and by the second charging of reservoir K_1 it had reached almost 100 ℃ (Supplementary Fig. 4).

After deposition, the early diagenesis had major influences on the supply of high-valence Mn in

the T_1b Formation after deposition. The increasing pressure of overlying sediments resulted in compaction, promoting the mechanical infiltration of clay-rich pore water and the formation of clay grain coatings. The clays are presently illite or mixed-layers I/S[6], although they are likely to have been more smectite-rich upon deposition. Dissolution of labile detrital grains, such as anorthite components in plagioclase, lithic fragments, and ferromagnesian grains, would have been possible at that time[31, 32]. Ferric iron derived from dissolution of iron-bearing grains or clay, precipitated as hematites through the dehydration of hydroxides, and reddening the rocks[31, 33, 34]. Meanwhile, the increasing formation temperature promoted the illitization of smectite in the I/S layers. The depletion of organic matter in the T_1b Formation meant that only weak decomposition of organic matter occurred, with little reduction of high-valence Mn and Fe oxides. This inference is supported by the abundant brown clastic rocks in T_1b Formation cores at depths of 2880–3900 m (Supplementary Fig. 2).

After charging by oil- and gas-bearing fluids, the high-valence Mn reacted to oxidize CH_4 to CO_2 at temperatures above 80 ℃. Organic acids and the generated CO_2 in petroleum-bearing fluids may also have promoted the dissolution of orthoclase[6], and the dissolution of detrital feldspars would have released substantial amounts of Ca^{2+} into the formation water. In some reservoir beds of the T_1b Formation, Mn-rich calcites then precipitated in the weakly alkaline fluid environment, along with kaolinite. During the two stages of reservoir oil and gas charging in the Jurassic and Cretaceous, respectively, the formation temperature of the latter was obviously higher than that of the former. As a higher temperature is favorable for the thermochemical oxidation of methane, more high-valence Mn could have been reduced to Mn^{2+} by methane during the later stage, with the Mn content of late-stage calcite cements thus being higher than that of early-stage calcites.

Supplementary Figures

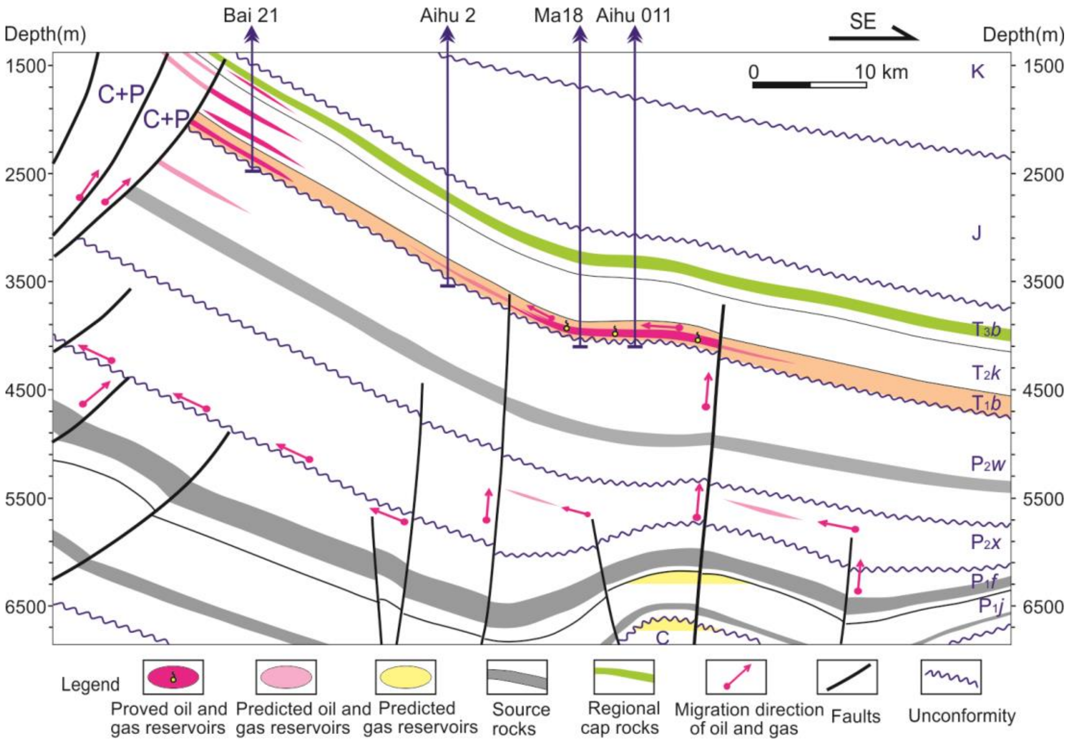

Supplementary Fig. 1. Typical oil and gas reservoir profile showing the combination of source rock, reservoir, and cap rocks in the study area. Oil and gas migrated into T_1b along steeply dipping faults.

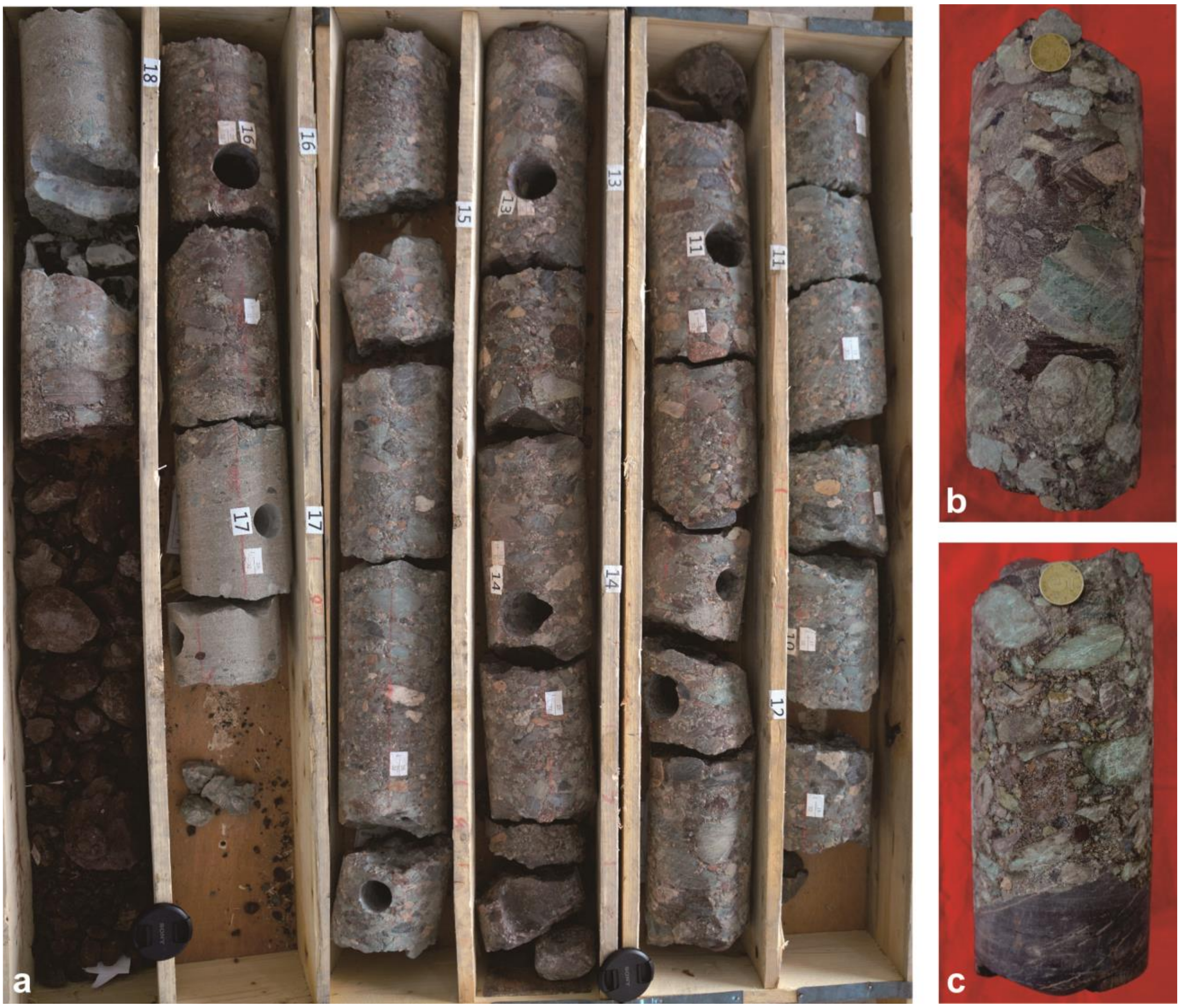

Supplementary Fig. 2. Deep burial T_1b cores that have not been reduced by hydrocarbon-bearing fluids. (a) Most of the pebbly conglomerates retain their original brown color of deposition; sandstone and conglomerates have locally been reduced and show a greyish-green colour. The diameter of the lens cap is 6 cm. Depths of 3850.8–3865.3 m in the T_1b_2 Formation, Well Aihu012. (b) Brown muddy intraclasts among pebbles in the conglomerate, and hematite in the clay-rich matrix. The diameter of the coin is 2 cm. Depth of 3842.5 m in the T_1b_2 Formation, Well Ma18. (c) Hematite in thin clay coatings around grains and in the clay-rich matrix in pebbly conglomerate. The brown color of the underlying mudstone reflects a high hematite content. Depth of 3853.7m in the T_1b_2 Formation, Well Ma18.

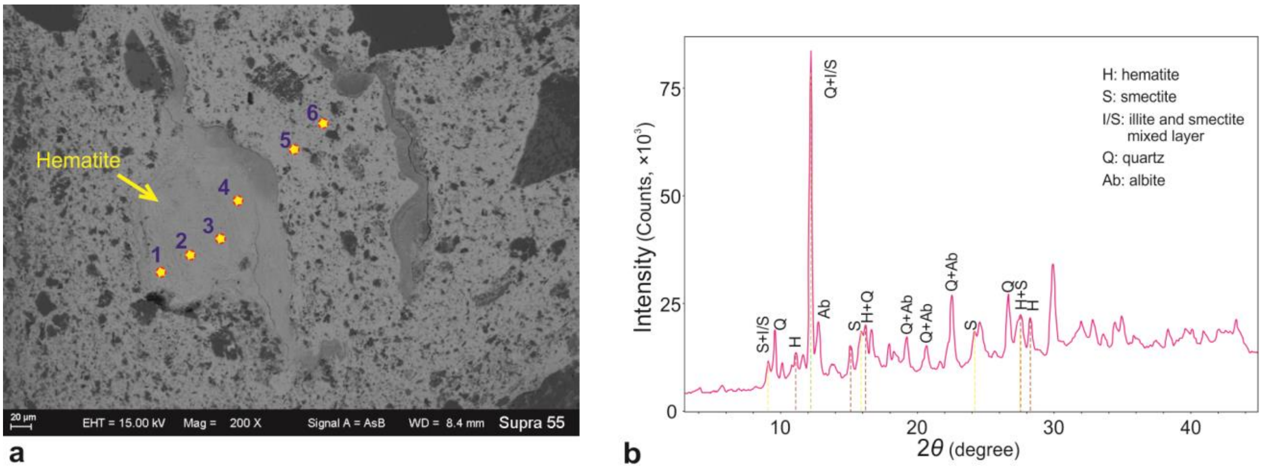

Spot No.	1	2	3	4	5	6
	hematite				hematite+smectite	
CaO	0.01	bdl	bdl	0.02	0.64	0.50
Na_2O	0.04	0.02	bdl	0.09	0.09	0.30
K_2O	0.04	0.01	0.02	0.06	1.23	3.56
TiO_2	1.16	2.05	1.99	3.03	3.68	1.03
MgO	0.10	0.03	bdl	0.01	0.53	2.95
Fe_2O_3	**95.49**	**95.16**	**94.25**	**91.44**	**60.26**	**41.64**
Al_2O_3	0.28	0.39	0.38	0.56	11.18	13.58
Mn_2O_3	**1.15**	**0.95**	**1.00**	**1.47**	**0.61**	**0.74**
SiO_2	1.21	1.30	1.27	1.94	20.44	28.30
Total	99.45	99.89	98.91	98.61	98.66	92.59

"bdl"denotes below detection limit.

c

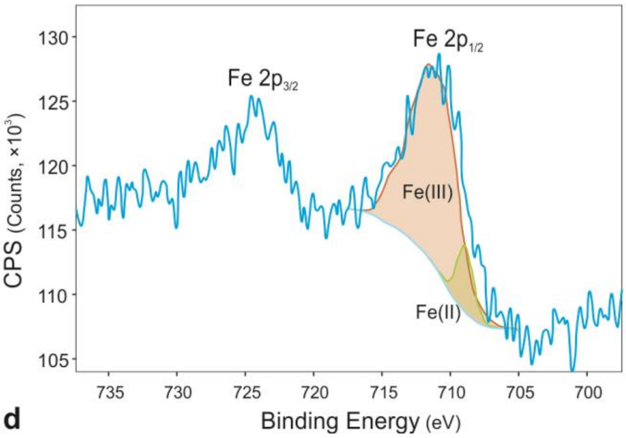

Supplementary Fig. 3. Minerals containing high-valence Mn in the T_1b Formation reservoir rocks. (a) Isolated hematite with a high Mn content and hematite disseminated in the clay-rich matrix. (b) XRD spectrum showing that abundant hematite is distributed in the smectite and I/S-rich matrix of the conglomerate. (c) The Mn content, shown as the Mn_2O_3 content of the isolated hematite, is clearly higher than that of the hematite disseminated in the clay. The data were obtained by EPMA. (d) XPS spectra indicate that the Fe is trivalent in the hematite of the brown rock. The Fe^{2+} signal is from ilmenite.

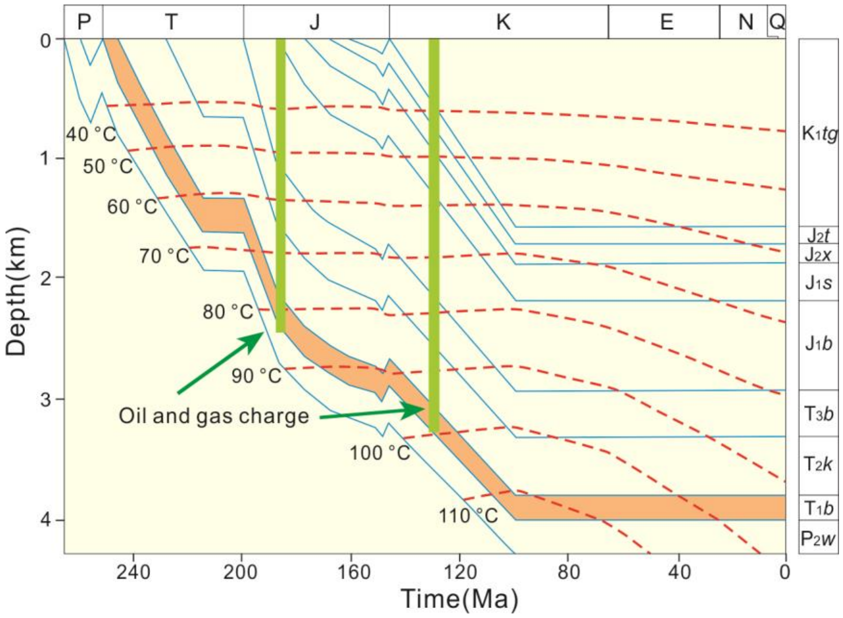

Supplementary Fig. 4. Burial history and thermal evolution of the Baikouquan Formation in the study area, taking Well Ma18 as an example.

Supplementary Tables

Supplementary Table 1 *In situ* $\delta^{13}C$ and $\delta^{18}O$ of calcite in T_1*b* reservoir rocks by SIMS

Sample No.	whole rock calcite $\delta^{13}C$ (‰, VPDB)	Spot No.	CaO (wt.%)	MgO (wt.%)	MnO (wt.%)	FeO (wt.%)	Ca/(Ca+Mg+Fe +Mn) (mol.%)	Mg/(Ca+Mg+Fe +Mn) (mol.%)	Mn/(Ca+Mg+Fe +Mn) (mol.%)	Fe/(Ca+Mg+Fe +Mn) (mol.%)	$\delta^{13}C$ (‰, VPDB)	1σ of $\delta^{13}C$	$\delta^{18}O$ (‰, VPDB)	1σ of $\delta^{18}O$
M-39	-42.29	1	50.79	0.27	6.24	0.02	90.51	0.68	8.79	0.02	-48.77	0.42	-20.47	0.25
		2	52.98	0.02	3.20	0.15	95.21	0.04	4.55	0.20	-44.61	0.42	-18.72	0.25
		3	52.50	0.01	4.32	0.10	93.73	0.03	6.09	0.14	-47.86	0.42	-18.75	0.25
		4	50.17	0.28	6.40	bdl	90.21	0.69	9.10	bdl	-43.97	0.42	-18.61	0.25
		5	50.17	0.28	6.40	bdl	90.21	0.69	9.10	bdl	-53.97	0.41	-19.95	0.25
2-M18-3	-57.46	1	52.02	0.03	6.61	0.30	90.44	0.08	9.08	0.40	-59.10	0.50	-21.29	0.20
		2	50.11	0.06	8.49	0.22	87.82	0.13	11.75	0.30	-59.27	0.49	-20.63	0.20
		3	52.40	0.05	6.16	0.19	91.17	0.11	8.46	0.26	-57.86	0.50	-20.95	0.20
		4	46.54	0.28	9.31	3.01	82.19	0.68	12.99	4.14	-56.32	0.49	-20.98	0.20
		5	47.05	0.24	9.12	2.81	82.86	0.59	12.69	3.86	-58.29	0.50	-20.94	0.20
		6	46.50	0.19	9.16	2.97	82.57	0.46	12.85	4.12	-60.65	0.49	-21.06	0.20

“bdl”denotes below detection limit.

Supplementary Table 2 Composition and C_1–C_4 $\delta^{13}C$ of natural gas from T_1b

Samples	Natural gas composition (%)													Gas dryness index	ERP	$\delta^{13}C$(‰, V-PDB)			
	O_2	N_2	CO_2	CH_4	C_2H_6	C_3H_8	nC_4H_{10}	iC_4H_{10}	nC_5H_{12}	iC_5H_{12}	C_6H_{14}	C_7H_{16}	C_8H_{18}			CH_4	C_2H_6	C_3H_8	C_4H_{10}
M139-G	bdl	3.82	0.37	90.39	2.82	0.79	0.44	0.35	0.28	0.22	0.34	0.13	0.06	0.9486	0.0039	-43.6	-29.5	-27.6	-26.5*
M154-G	bdl	1.38	0.43	92.74	2.84	0.83	0.45	0.37	0.30	0.24	0.33	0.09	0.02	0.9486	0.0044	-43.3	-28.8	-27.7	-28.6
MH1-G	bdl	1.23	0.12	78.9	9.97	4.95	1.51	1.86	0.60	0.56	0.29	0.01	bdl	0.8022	0.0012	-46.0	-30.8	-29.7	-28.8
M13-G	bdl	2.56	0.12	92.18	3.04	0.83	0.36	0.28	0.18	0.14	0.19	0.08	0.04	0.9502	0.0012	-42.9	-29.2	-27.4	-26.4
AH013-G	0.67	6.47	1.46	70.23	7.70	5.23	2.74	2.39	1.07	0.93	0.85	0.26	bdl	0.7778	0.0163	-40.4	-31.4	-30.2	-29.4
M134-G	bdl	3.02	0.32	89.9	3.18	1.01	0.57	0.49	0.37	0.34	0.56	0.18	0.06	0.9378	0.0034	-42.0	-27.5	-27.2	-26.0
M18-G-1	bdl	2.19	0.78	86.08	5.23	2.29	1.17	0.97	0.39	0.37	0.34	0.11	0.09	0.8920	0.0081	-42.8	-30.8	-28.6	-27.7
M18-G-2	bdl	3.15	0.87	87.34	4.30	1.78	0.84	0.68	0.30	0.31	0.31	0.09	0.02	0.9141	0.0091	-43.0	-30.8	-28.7	-27.5
M18-G-3	bdl	1.52	0.13	91.33	3.72	1.30	0.63	0.52	0.28	0.25	0.27	0.04	bdl	0.9317	0.0013	/	/	/	/
M6-G	bdl	4.85	0.06	85.77	5.07	1.75	0.74	0.79	0.33	0.40	0.28	0.02	bdl	0.9043	0.0006	-46.8	-31.9	-28.7	-28.7
AH011-G	bdl	3.14	0.22	85.93	5.06	2.18	1.09	0.97	0.47	0.42	0.38	0.15	bdl	0.8940	0.0023	-43.1	-31.0	-29.2	-28.3
AH1-G	bdl	4.8	1.87	70.65	7.99	5.2	2.93	2.58	1.31	1.16	1.17	0.33	0.01	0.7694	0.0205	-39.3	-30.9	-29.5	-29.0

a) "bdl"indicates below the detection limitation; b) "/"denotes not carrying on analysis on the sample; c) Gas dryness index = $C_1/(C_{1-5})$; d) Extent of thermochemical iron and manganese oxides reduction parameter (ERP)=$CO_2/[CO_2+ \sum nC_nH_{2n+2}]$, referred from Krouse et al., 1988.

Supplementary Table 3 Typical biomarker parameters and whole oil, alkane, and arene $\delta^{13}C$ for oil from T_1b

Samples	Oil density (20 ℃)	CPI	Pr/Ph	Pr/nC_{17}	C_{20}TT%	C_{21}TT%	C_{23}TT%	C_{21-}/C_{22+}	C_{29} sterane 20S/(20S+20R)	C_{29} sterane ββ/(αα+ββ)	Ts/Tm	Whole oil $\delta^{13}C$	Alkane $\delta^{13}C$	Arene $\delta^{13}C$
AH013-O	0.8395	1.08	1.05	0.90	33.87	32.8	33.33	1.65	0.52	0.73	0.57	-29.1	-29.5	-28.8
AH5-O	0.8076	1.09	1.24	0.71	41.59	30.27	28.14	2.73	0.50	0.65	0.33	-28.2	-28.2	-28.4
AH2-O	0.8299	1.07	1.03	0.82	34.56	33.19	32.24	1.91	0.48	0.61	0.44	-28.9	-29.1	-28.4
AH6-O	0.8242	1.09	1.09	0.88	36.49	32.85	30.66	2.19	0.51	0.71	0.48	-29.0	-29.6	-29.2
AH012-O	0.8368	1.10	1.08	0.90	35.91	31.11	32.76	2.22	0.45	0.64	0.24	-28.6	-28.6	-27.6
M18-O-1	0.8237	1.08	1.10	0.90	35.25	30.59	34.16	2.32	0.48	0.67	0.43	-28.7	-28.9	-27.8
M18-O-2	0.8058	1.06	1.08	0.88	34.88	31.26	33.86	2.51	0.48	0.69	0.41	-28.8	-29.1	-28.0
AH1-O	0.8269	1.06	1.13	0.89	35.95	32.08	31.97	2.17	0.49	0.7	0.40	-28.8	-28.9	-28.6
AH011-O	0.8374	1.07	1.07	0.92	34.17	33.02	32.81	1.61	0.49	0.64	0.24	-28.6	-28.9	-28.2

$\delta^{13}C$ values are reported as per mil (‰) relative to the V-PDB standard.

Supplementary Table 4 Bulk rock major elements content by XRF for main rock types in T_1*b*

Member	Sample	Lithology	SiO_2 (wt,%)	TiO_2	Al_2O_3	$Fe_2O_3T^a$	$Mn_2O_3T^b$	MgO	CaO	Na_2O	K_2O	P_2O_5	LOI	TOTAL
T_1b_3	AH4-7-2	silty mudstone	70.01	0.48	14.22	5.26	0.17	0.97	0.83	2.25	2.82	0.14	3.08	100.23
	MX1-1		63.47	0.81	16.89	6.88	0.13	1.45	0.78	2.41	3.43	0.07	3.38	99.70
T_1b_2	M606-1		64.85	0.72	15.64	6.60	0.26	1.61	0.82	1.82	3.65	0.06	4.19	100.22
	M18-12		61.14	0.85	19.81	5.24	0.13	1.51	0.88	1.18	5.04	0.02	4.15	99.95
T_1b_1	M18-24		62.4	0.94	19.34	4.15	0.11	1.33	1.14	1.51	4.12	0.12	4.3	99.47
	M18-26		62.47	0.88	17.55	7.57	0.12	1.49	0.98	1.13	3.63	0.12	4.05	99.99
	AH4-1-2	sandstone	72.44	0.42	12.31	5.51	0.13	0.92	0.68	2.38	2.80	0.11	2.34	100.05
T_1b_3	AH4-5-2		72.43	0.56	13.01	4.60	0.12	0.74	0.68	2.35	3.00	0.10	2.37	99.96
	MX1-2		67.25	0.84	15.66	6.17	0.22	1.59	0.57	2.62	2.95	0.05	1.79	99.71
T_1b_2	AH1-4-2		77.6	0.37	11.13	2.88	0.12	0.90	0.58	2.85	1.68	0.08	2.56	100.75
	M18-5		59.74	0.62	21.12	5.56	0.16	1.33	0.66	1.66	5.33	0.01	3.47	99.66
T_1b_1	AH1-17		77.62	0.53	11.00	3.53	0.12	0.54	0.76	1.93	1.89	0.01	2.56	100.48
	AH4-8-2	Conglomerate	69.41	0.62	13.63	6.56	0.07	0.79	0.78	1.88	3.20	0.07	2.98	99.99
T_1b_3	AH4-6		67.19	0.68	14.48	8.07	0.08	0.79	0.79	1.63	3.18	0.08	3.38	100.34
	MX1-4		71.99	0.57	13.20	4.71	0.16	1.03	0.77	2.67	1.94	0.12	2.58	99.74
	X1-10		72.24	0.48	13.21	4.98	0.19	0.89	0.71	2.54	2.31	0.08	2.58	100.21
T_1b_2	X1-15		69.07	0.48	12.81	8.69	0.27	0.97	0.74	1.95	2.47	0.07	2.94	100.46
	M-6		58.04	0.51	16.43	12.71	0.48	1.97	0.74	2.46	2.44	0.12	4.75	100.66

a) Fe_2O_3 T, total iron as Fe_2O_3., b) Mn_2O_3T, total manganese as Mn_2O_3. LOI is the weight loss on ignition.

Supplementary Table 5 Rock samples information from the Baikouquan Formation

Lithology	Details	Sample number
Muddy pebble conglomerate	The rock is mainly composed by 10~30 mm pebbles, with >10 % clay, few containing coarse grain calcite.	37
Sandy granule conglomerate	Gravel content is >50 %, whose diameter is 2~6 mm; the matrix inter gravels are mainly sands and the clay of 8 %, containing various content of calcite cements.	45
Sandstone	Deposition of braided stream or subaqueous channels, and cross beddings are common.	16
Silty mudstone	The rock is brown or grey, containing 15~25 % silt.	5

Supplementary Table 6 Oil and gas samples information from the Baikouquan Formation

Types	Member	Sample No.	Well No.	Depth (m)	Types	Member	Sample No.	Well No.	Depth (m)
Oil	T_1b_3	AH5-O	Aihu5	3796~3800	Gas	T_1b_3	M154-G	Ma154	3026~3037
		AH013-O	Aihu013	3798~3816			M13-G	Ma13	3106~3129
	T_1b_2	AH2-O	Aihu2	3310~3336		T_1b_2	M134-G	Ma134	3169~3188
		AH6-O	Aihu6	3878~3909			M18-G-1	Ma18	3854~3871
		AH012-O	Aihu012	3839~3864			M18-G-2	Ma18	3854~3871
		M18-O-1	Ma18	3854~3871		T_1b_1	M18-G-3	Ma18	3898~3920
	T_1b_1	M18-O-2	Ma18	3898~3920			M6-G	Ma6	3871~3880
		AH1-O	Aihu1	3848~3862			AH011-G	Aihu011	3848~3882
		AH011-O	Aihu011	3848~3882			AH013-G	Aihu013	3798~3816
Gas	T_1b_3	MH1-G	Mahu1	3284~3310			AH1-G	Aihu1	3848~3862
		M139-G	Ma139	3261~3277					

Supplementary References

1. Chen, F. J., Wang, X. W. & Wang, X. W. Prototype and tectonic evolution of the Junggar basin, northwestern China. *Earth Sci. Front.* **12**, 77–89 (2005).
2. Cao, J. et al. The Permian hybrid petroleum system in the northwest margin of the Junggar Basin, northwest China. *Mar. Petrol. Geol.* **22**, 331–349 (2005).
3. Carroll, A. R. et al. Junggar Basin, northwest China: Trapped Late Paleozoic ocean. *Tectonophysics* **181**, 1–14 (1990).
4. Cai, Z. X., Chen, F. J. & Jia, Z. Y. Types and tectonic evolution of Junggar Basin. *Earth Sci. Front.* **7**, 431–440 (2000).
5. Ablimit, I. et al. Accumulation mechanism and controlling factors of the continuous hydrocarbon plays in the Lower Triassic Baikouquan Formation of the Mahu Sag, Junggar Basin, China. *Nat. Gas Geosci.* **1**, 309–318 (2016).
6. Kang, X. et al. Selective dissolution of alkali feldspars and its effect on Lower Triassic sandy conglomerate reservoirs in the Junggar Basin, northwestern China. *Geol. J.* **53**, 475–499 (2018).
7. Kuang, L. C., Tang, Y., Lei, D. W., Wu, T. & Qu, J. H. Exploration of fan-controlled large-area lithologic oil reservoirs of Triassic Baikouquan Formation in slope zone of Mahu Sag in Junggar Basin. *Chn Petrol. Explor.* **19**, 14–23 (2014).
8. Qi, W. et al. Fluid inclusion and hydrocarbon charge history for reservoir of Baikouquan Formation in the Mahu Sag, Junggar Basin. *Nat. Gas Geosci*. **26**, 64–71 (2015).
9. Chen, Y. B. et al. Characteristics of fault evolution in Mahu slope area of Junggar Basin and its implications to the reservoir in the Lower Triassic Baikouquan Formation. *Nat. Gas Geosci.* **26**,11–24 (2015).
10. Jia, H. et al. Retreating fan-delta system in the northwestern Junggar Basin, northwestern China—Characteristics, evolution and controlling factors. *J. As. Earth Sci.* **123**, 162–177 (2016).
11. Zhang, S. C. et al. Genetic analysis of the high quality reservoir of the Triassic Baikouquan Formation in Mabei Region, Junggar Basin. *Acta Sediment. Sin.* **32**, 1171–1180 (2014).
12. Eugster, H. P. & Jones, B. F. Behavior of major solutes during closed-basin brine evolution. *Am. J. Sci.* **279**, 609–631 (1979).
13. Schmid, S., Worden, R. H. & Fisher, Q. J. Carbon isotope stratigraphy using carbonate cements in the Triassic Sherwood Sandstone Group: Corrib Field, west of Ireland. *Chem. Geol.* **225**, 137–155 (2006).
14. Thompson, J. & Meadows, N. S. Clastic sabkhas and diachroneity at the top of the Sherwood Sandstone Group: East Irish Sea Basin. *Geol. Soc. Lond. Spec. Publ.* **124**, 237–251 (1997).
15. Walanda, D. K., Lawrance, G. A. & Donne, S. W. Hydrothermal MnO_2: synthesis, structure, morphology and discharge performance. *J. Power Sourc.* **139**, 325–341 (2005).
16. Retallack, G. J. et al. Multiple Early Triassic greenhouse crises impeded recovery from Late Permian mass extinction. *Palaeogeogr. Palaeoclim. Palaeoecol.* **308**, 233–251 (2011).
17. Sun, Y. et al. Lethally hot temperatures during the Early Triassic greenhouse. *Science* **338**, 366–370 (2012).
18. Shan, X. et al. Provenance analysis of Triassic Baikouquan Formation in the area around Mahu Depression, Junggar Basin. *Acta Sediment. Sin.* **34**, 930–939 (2016).
19. Galakhov, V. R. et al. Mn 3*s* exchange splitting in mixed-valence manganites. *Phys. Rev. B* **65**, 1–4 (2002).

20. Ilton, E. S., Post, J. E., Heaney, P. J., Ling, F. T. & Kerisit, S. N. XPS determination of Mn oxidation states in Mn (hydr) oxides. *Appl. Surf. Sci.* **366**, 475–485 (2016).

21. Haynes, W. M. *CRC handbook of chemistry and physics* Ch. 5 (CRC press, Boca Raton, 2014).

22. Yang, S. et al. Elemental mercury capture from flue gas by magnetic Mn–Fe spinel: effect of chemical heterogeneity. *Industr. Engineer. Chem. Res.* **50**, 9650–9656 (2011).

23. Alvarez, M., Rueda, E. H. & Sileo, E. E. Simultaneous incorporation of Mn and Al in the goethite structure. *Geochim. Cosmochim. Acta* **71**, 1009–1020 (2007).

24. Manceau, A. et al. Crystal chemistry of trace elements in natural and synthetic goethite. *Geochim. Cosmochim. Acta* **64**, 3643–3661 (2000).

25. Liu, H. et al. Geochemical fates and unusual distribution of arsenic in natural ferromanganese duricrust. *Appl. Geochem.* **76**, 74–87 (2017).

26. Singh, B., Sherman, D. M., Gilkes, R. J., Wells, M. A. & Mosselmans, J. F. W. Incorporation of Cr, Mn and Ni into goethite (α-FeOOH): mechanism from extended X-ray absorption fine structure spectroscopy. *Clay Miner.* **37**, 639–649 (2002).

27. Guillaume, D. et al. Experimental synthesis of chlorite from smectite at 300 ℃ in the presence of metallic Fe. *Clay Miner.* **38**, 281–302 (2003).

28. Artamonova, I. V., Gorichev, I. G. & Godunov, E. B. Kinetics of manganese oxides dissolution in sulphuric acid solutions containing oxalic acid. *Engineering* **5**, 714–719 (2013).

29. Wolela, A. M. & Gierlowski-Kordesch, E. H. Diagenetic history of fluvial and lacustrine sandstones of the Hartford Basin (Triassic-Jurassic), Newark Supergroup, USA. *Sediment. Geol.* **197**, 99–126 (2007).

30. Armstrong, F. A. Why did Nature choose manganese to make oxygen? *Philos. Trans. R. Soc. Lond. B* **363**, 1263–1270 (2008).

31. Qiu, N. S., Yang, H. B. & Wang, X. L. Tectono-thermal evolution in the Junggar Basin. *Chn. J. Geol.* **37**, 423–429 (2002).

32. Greenwood, P. J. & Habesch, S. M. Diagenesis of the Sherwood Sandstone Group in the southern East Irish Sea Basin (Blocks 110/13, 110/14 and 110/15): constraints from preliminary isotopic and fluid inclusion studies. *Geol. Soc. Lond. Spec. Publ.* **124**, 353–371 (1997).

33. Burley, S. D. Patterns of diagenesis in the Sherwood Sandstone Group (Triassic), United Kingdom. *Clay Miner.* **19**, 403–440 (2015).

34. Olivarius, M. et al. Diagenetic effects on porosity–permeability relationships in red beds of the Lower Triassic Bunter Sandstone Formation in the North German Basin. *Sediment. Geol.* **321**, 139–153 (2015).
