# Supplementary material for: Thermochemical oxidation of methane induced by high-valence metal oxides in a sedimentary basin
Source: Nat Commun. 2018 Dec 3;9:5131. doi: 10.1038/s41467-018-07267-x (PMC6277441; doi:10.1038/s41467-018-07267-x)
Supplement: Supplementary file 3 — Description of Additional Supplementary Files [file 41467_2018_7267_MOESM3_ESM.pdf]

Description of Additional Supplementary Files

Supplementary Data 1

Description: Major elements contents by EPMA for calcites in T_1*b* sandy conglomerates.

Supplementary Data 2

Description: $\delta^{13}C$ and $\delta^{18}O$ of calcites in T_1*b* reservoir rocks and P_2*w* source rocks
